# Effects of Inhibition of IKK Kinase Phosphorylation On the Cellular Defence System and HSP90 Activity

**DOI:** 10.1007/s10753-023-01894-3

**Published:** 2023-08-29

**Authors:** Miriam Giacomarra, Martina La Torre, Giovanna Montana

**Affiliations:** 1 Istituto di Ricerca e Innovazione Biomedica, Consiglio Nazionale delle Ricerche (CNR), Via Ugo La Malfa 153, 90146 Palermo, Italy; 2 Consiglio Nazionale delle Ricerche (CNR), Istituto di Farmacologia Traslazionale, 00133 Rome, Italy

**Keywords:** IKK kinase, inflammation, anti-oxidative pathway, HSP90, Keap1

## Abstract

The present study was conceived to examine the effects of inhibition of BMS-345541 mediated IKK kinase phosphorylation on the cellular defence system as well as on anti-inflammatory response and HSP90 activity. The analysis was conducted in A549 cell line, since such cells carry a homozygous Keap1 mutation (G333C) that alters its interaction with Nrf2. Recent data have highlighted that Keap1, HSP90 protein and IKK kinase interact reciprocally and particularly Keap1 protein is involved in HSP90 and anti-oxidative pathway regulation. The activities of COX2 and HO1 were investigated by real time and immunoblot analysis along with the synthesis and activity of inducible forms of heat shock protein HSP90. Pre-treatment with IKK kinase inhibitor proved to be a protective means to lower the activity of inflammatory cascade, so preventing the formation of excessive amounts of pro-inflammatory molecules. The inhibitor of IKK kinase BMS-345541 was added to cultured A549 cells before the Escherichia coli lipopolysaccharide (LPS) addition. The viability of the cells was determined after 1–24 h incubation with BMS-345541 at concentrations ranging from 1,25–5 µM. It was found that 1 µM concentration does not significantly affected cell viability (data not shown). As a result, the treatment with 1 μM of BMS-345541 induces the inhibition of IKK phosphorylation. In the A549 cells treated with BMS-345541 and LPS, COX2 activity is not induced: mRNA and protein levels have not increased, while there is an increase in the level of HSP90, HO1 proteins and mRNA. The results suggest that the IKK inhibition is effective in the reduction of the inflammatory response thanks to mechanisms involving both the heat shock cellular defense system and the antioxidative pathway.

**Graphical Abstract:**

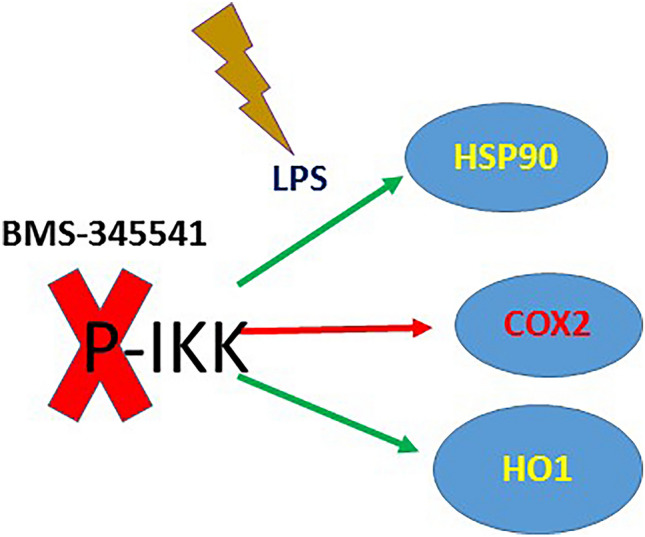

## INTRODUCTION

In the last 20 years the knowledge about the NF-kB/IKK role in the most important cellular processes such as inflammation, tumorigenesis and cancer progression has been very impressive. In particular, it has become clear that such processes are closely linked and interconnected [1–3]. In mammals, the NF-kB complex consists of five monomeric proteins (p65 / RelA, RelB, cRel, p50 and p52) forming homodimers or hetero-dimers that bind with different affinity to DNA. The “canonical” regulation of NF-kB is intricate and mediated by a protein kinase complex, consisting of a NEMO scaffold / adapter protein (IKKγ) and two IκB kinases (IKKα and IKKβ); for this reason, it is also called NEMO dependent. IKKα is an 85 kDa protein, initially identified as a serine-threonine with unknown function. IKKβ is an IKKα-related 87 kDa protein. IKKγ (50–52 kDa) contains several regions with spiral wound N-terminal helical motifs, an LZ and a C-terminal Zn-finger. IKK kinases are activated through the phosphorylation of serines located in an activation T-loop with a complicated mechanism which has not been fully understood yet. This phosphorylation probably involves a conformational change, responsible for the activation of kinases. In condition of cellular homeostasis, NF-kB is kept inactive in the cytoplasm through interaction with inhibitory molecules belonging to the IkB family [4, 5]. When IkB is phosphorylated and polyubiquitinated, it is degraded by proteasome, so allowing the release of NF-kB which can translocate into the nucleus and activate the transcription of its target genes, binding to DNA sequences called response elements (RE). The formation of the DNA / NF-kB complex involves other proteins as coactivators, thus allowing the activation and transcription of target genes. Moreover, the IKK complex has a key role as signaling hub for NF-kB activation and as an interface with other signaling cascade, such as mTOR and MAPK pathways [6, 7]. Several studies have pointed out that IKKβ is involved in promoting tumorigenicity through the inhibition of tumor suppressors by phosphorylation; therefore, IKKβ is considered an oncogenic kinase [5, 8]. Recently, it was highlighted that IKK complex interacts with the Keap1 protein, the master repressor of Nrf2 [9–12]. KEAP1 and NRF2 are the two key genes that regulate the oxidative stress pathway. Nuclear factor erythroid 2–related factor 2 (NFE2L2 or NRF2) is considered as one of the major antioxidant transcription factors against oxidative and electrophilic stress [13, 14]. When cells are exposed to oxidative stress, KEAP1 undergoes conformation changes and dissociation from NRF2 [15]. Nrf2, released from the Keap1–Nrf2 complex, translocates from the cytoplasm into the nucleus and activates the expression of related antioxidant genes [16]. Moreover, Keap1 works as a E3 ligase of IKKβ, since it has an ETGE-Motif-NQE36TGE39- homologous to the one present in the Nrf2 protein, so KEAP1 is considered as a IKKβ interacting protein [17] and mutations or alterations in Keap1 gene are correlated with cancer or a deeply altered cell stress response [18]. A recent study [9, 10] carried out in RAW264.7 cells shows that IKK kinase is activated by canonic LPS-signalling when Keap1 is functionally active. Keap1 is a key protein in the interaction between inflammation and oxidative stress. Interestingly, it emerges that Hsp90 and KEAP1 interact upon heat shock, leading to the activation of NRF2 [19], and that the environmental redox changes can induce heat shock genes [20, 21]. Therefore, in this paper we analyze the crosstalk between IKK kinase, HSP90 and anti-inflammatory response mediated by COX2 and Heme Oxygenase-1 (HO1) enzymes. More precisely, we induce the inhibition of IKK phosphorylation by using BMS-345541 in the cell line A549 mutated in Keap1 allele [22] in order to observe the effects on the underlying molecular mechanism.

## MATERIALS AND METHODS

### Cell Culture and Reagents

A549 cell line (provided by Francesca Sardina and Cinzia Rinaldo (IBPM-CNR, Roma, Italy) was cultured in Dulbecco’s Modified Eagle Medium (DMEM) supplemented with 10% v/v heat-inactivated fetal bovine serum (FBS) and antibiotics (100 U/ml penicillin and 100 μg/ml streptomycin) at 37 °C in a humidified atmosphere with 5% CO_2_. FBS, DMEM, penicillin and streptomycin (10,000 U/ml) were purchased from GIBCO (Grand Island, NY). LPS from E. coli serotype O55:B5 was purchased from Sigma-Aldrich, Inc. (St. Louis, MO). TRIzol was purchased from Invitrogen, the QuantiNova RT PCR kit from Hilden, Germany, and BrightGreen 2X qPCR MasterMix-ROX from ABM (Canada). Nitrocellulose blotting membrane was purchased from Amersham Protran (Buckinghamshire, UK). BMS- 345,541 was purchased from Sigma-Aldrich, Inc. (St. Louis, MO).

### RT-qPCR

A549 cells were cultured (1 × 10^6^ cells/well) in a 6-well plate overnight in DMEM supplemented with 10% bovine serum. Cells were treated with 100 ng/ml LPS or without (negative control), in the presence or absence of 1 μM BMS- 345,541. Cells stimulated with 100 ng/ml LPS for 4 h served as a positive control. After 4 h of stimulation, the cells were detached from the wells and washed once with PBS. Total RNA was isolated with TRIzol according to the manufacturer’s instructions and was quantified by UV absorbance spectrophotometry and reverse transcribed with QuantiNova RT PCR kit. QPCR was performed in triplicate on each cDNA sample for each gene by using BrightGreen 2X qPCR MasterMix-ROX. The Quantitect primers set purchased from Qiagen are:

Hsp90 NM005348.2 F: 5′-CGA TGA ATA TGC CAT GAC T-3′

R: 5′-TCC ATA GCA GAT TCT CCA G-3′

COX2 GI:31,981,524 F:5′-CAG ACA ACA TAA ACT GCG CCTT-3′

R: 5′-GAT ACA CCT CTC CAC CAA TGACC -3′

HO1 NM_010442.1 F: 5’-CACTCTGGAGATGACACCTGAG-3’

R: 5’- GTGTTCCTCTGTCAGCATCACC-3’

HPRT NM194 F:5′-GCT ATA AAT TCT TTG CTG ACC TGC TG-3′

R: 5′-AAT TAC TTT TAT GTC CCC TGT TGA CTGG-3′

The threshold cycle (CT) values were calculated in relation to the housekeeping gene Hprt. In order to report the results, all data were normalized to Hprt, which was assigned an arbitrary expression level of 10,000, and relative gene expression values were calculated by the following formula: relative expression 10,000/2 CT, where CT is gene CT/Hprt CT. According to Applied Biosystems Real-Time PCR Software.Melting curve analysis was conducted to verify the purity and size of the resultant PCR products. At least three distinct biological samples were examined for each gene and treatment (each one performed in triplicate).

### Western Blotting

A549 cells (1 × 10^7^ cells) were cultured in 10-cm dishes (Falcon) and allowed to adhere for 24 h. After treatment with BMS- 345,541 1 μM for 1 h, followed by incubation with LPS 100 ngr/ml for 4 h, the cells were washed twice with cold PBS. Whole-cell lysates were obtained using RIPA buffer (Cell Signaling Inc. Beverly, MA, USA). The protein concentration of cell lysates was determined by the Bradford method. An amount of protein (30 μg) was separated on 8–16% Tris–Glycine Gel (BioRad) gels by electrophoresis and transferred to a nitrocellulose membrane. The membranes were subsequently incubated for 1 h at room temperature with 3% BSA in TBS buffer (0.1% v/v) to block non-specific binding and incubated with an appropriate primary antibody in 1% BSA in TBST (tween 0.01% v/v). Antibodies polyclonal antimouse recognizing HSP90, IKKα/β, COX2, HO1 and β-actin were purchased from Santa Cruz Biotechnology (Santa Cruz, CA, USA); antibody polyclonal antirabbit recognizing p-IKKα/β was purchased from Cell Signaling Technology Massachussetts, USA. Incubation with the secondary antibodies Alexa Fluor 680 goat anti-rabbit (1:2000) and Alexa Fluor 800 rabbit anti-mouse (1:5000) (Molecular Probes, Life Technologies, Carlsbad, CA, USA) was performed for 1 h at room temperature. Densitometry analysis was conducted using the Odyssey Infrared Imaging System (LiCOR Bioscience, NE, USA).

### Statistics

Statistical analysis. All data were analyzed by the one-way analysis of variance (one-way ANOVA) compared with the respective control group, followed by the multiple comparison test of Tukey’s, by using the OriginPro 7.5 statistical program with the level of significance set to P < 0.05. Each result is reported as the mean of three independent replicate experiments ± SE.

## RESULTS

### BMS-345541 Induces Depletion of Phosphorylation of IKK Kinase in LPS Treated A549 Cells

**Fig. 1:**
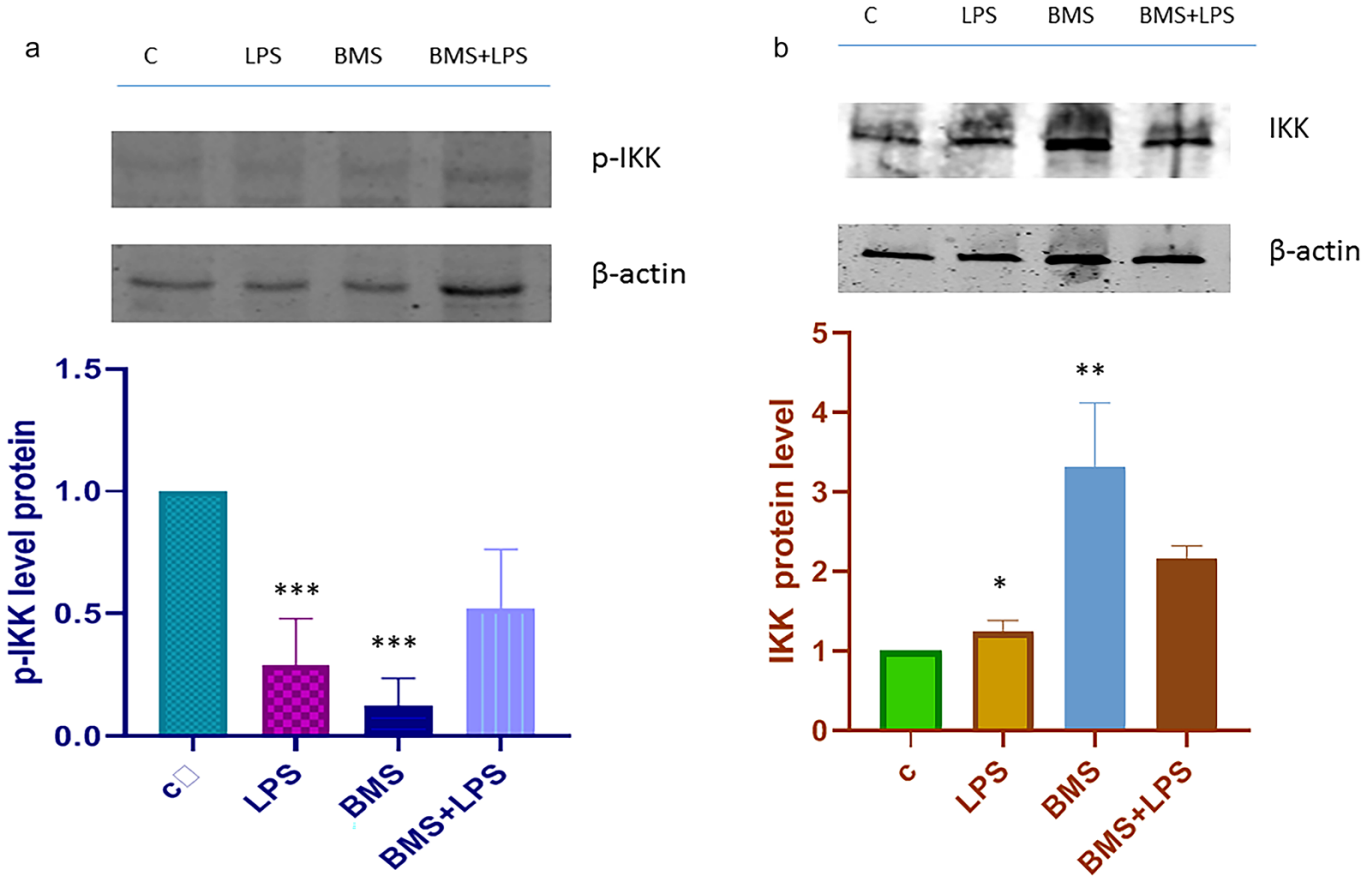
**a** Immunoblot analysis of p-IKK α/β. A549 cells were treated with or without BMS-345541 1 μM for 24 h, then treated with LPS 100 ngr/ml for 4 h. The whole cell lysates were obtained and analyzed with anti-p-IKKα/β antibody (Ser176/180 CellSignaling). Representative immunoblotting shows results for the protein levels of p-IKKα/β. Histogram is representative of the means ± SD of three replicates after normalization with actin. Protein levels are reported in arbitrary units as fold increase or decrease compared to control that is set to 1. Asterisks (***) indicate significant differences among groups (*** p < 0.001), **b** Immunoblot analysis of IKK α/β in A549 cells. A549 cells were treated as described above. Non-stimulated cells worked as control. The whole cell lysates were obtained and analyzed with anti IKK antibody (C-20, Santa Cruz Biotechnology). The relative protein levels of IKK are shown in the histogram. The data shown represents three independent experiments. β-actin immunolabelling was used as a normalization control. Data are reported as the mean ± SD; asterisks (*) indicate LPS vs control; asterisks (**) indicates BMS vs C $(\ast p<0.05;p<0.01)$.

The IKK kinase is the major activator of the NF-κB complex. The IKK complex consists of two catalytic subunits (IKKα and IKKβ) and one regulatory subunit (IKKγ/NEMO). Over the past two decades many studies have focused on the design of protein kinase inhibitors as potential drug targets and pharmacologic agents… A549 cells were exposed to 100 ng/ml LPS for 4 h; one hour before LPS, cells were treated with BMS-345541 1 µM, while non-treated cells worked as negative control. Total extracts were prepared and analyzed in immunoblotting for IKKα/β. As reported in Fig. 1a, when BMS-345541 treatment occurs, IKK phosphorylation is strongly inhibited. It is well known that LPS is a powerful phosphorylation activator of IKK kinase both *in vivo* and *in vitro*: in our study model the protein level of p-IKK after the BMS-345541 treatment, though in presence of LPS, has significantly decreased. Our findings are in line with the literature data demonstrating that BMS-345541 suppresses the LPS-induced pro-inflammatory response, as it induces depletion of phosphorylation of IKK complex. We tested that BMS-345541treatment also in A549 lung carcinoma cell line is able to deplete IKK activity. Figure 1b shows the immunoblot control for IKK kinase.

### Effects of IKK Phosphorylation Inhibition On Antioxidant Pathway Activity in A549 Cell Line

**Fig. 2:**
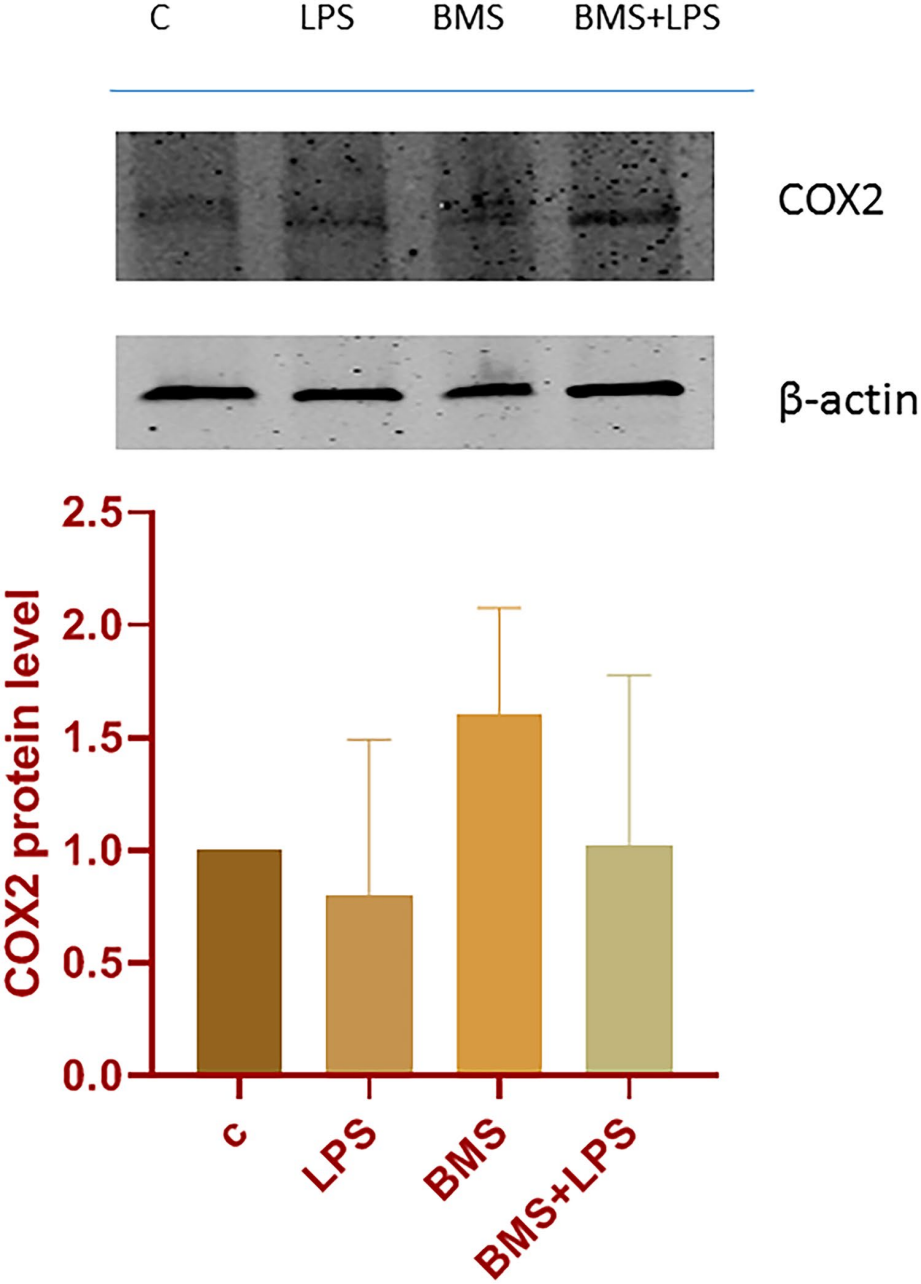
Immunoblot analysis of COX2 protein in A549 cell line. A549 cells were treated as described above. Total proteins were extracted and immunoblot analysis of COX2 protein level was effectuated. Densitometry analysis is shown in the graph; there are no significant differences among groups.

The transcriptional activation of the antioxidant-responsive element (ARE) plays a pivotal role in activating a defense mechanism against electrophiles and reactive oxygen species (ROS) toxicity. Nrf2 is a Cap-N-Collar transcription factor acting as a very effective signal transducer to respond to electrophiles and ROS by regulating transcription depending on ARE. The ARE-regulated genes comprise detoxification enzymes and antioxidant genes which provide protection against oxidative insults. COX enzymes exist in two isoforms, COX1, and COX2. COX1 is constitutively expressed in many tissues and is responsible for supporting normal physiologic levels of prostaglandins. The inducible COX2 isoform generates prostaglandins linked to inflammation progression and pain. In this study, we focused on the effect of IKK kinase inhibition BMS- 345,541 mediated on the COX2 activity at various biological levels including transcripts and proteins in A549 cells, by exposing cells to a highly selective inhibitor of the catalytic subunits of IKKα and IKKβ-namely BMS-345541 (1 µM concentration). Total protein and RNA were extracted from A549 cells exposed to LPS (100 ng/ml) for 4 h; after 1 h BMS- 345,541 exposure, the cells underwent LPS and results from immunoblot and qPCR were compared to those obtained from BMS- 345,541 unexposed cells. The analysis of COX2 protein levels did not show significant changes among each group (see Fig. 2). Similarly, the amplification of cDNA with specific primers for COX2 and HPRT as an internal standard showed that the transcription of COX2 mRNA did not even increase in LPS-treated A549 cells (data not shown). These results highlight that the in the A549 cells treated with BMS- 345,541 does not induce a proinflammatory response mediated by the activation of COX2.

**Fig. 3:**
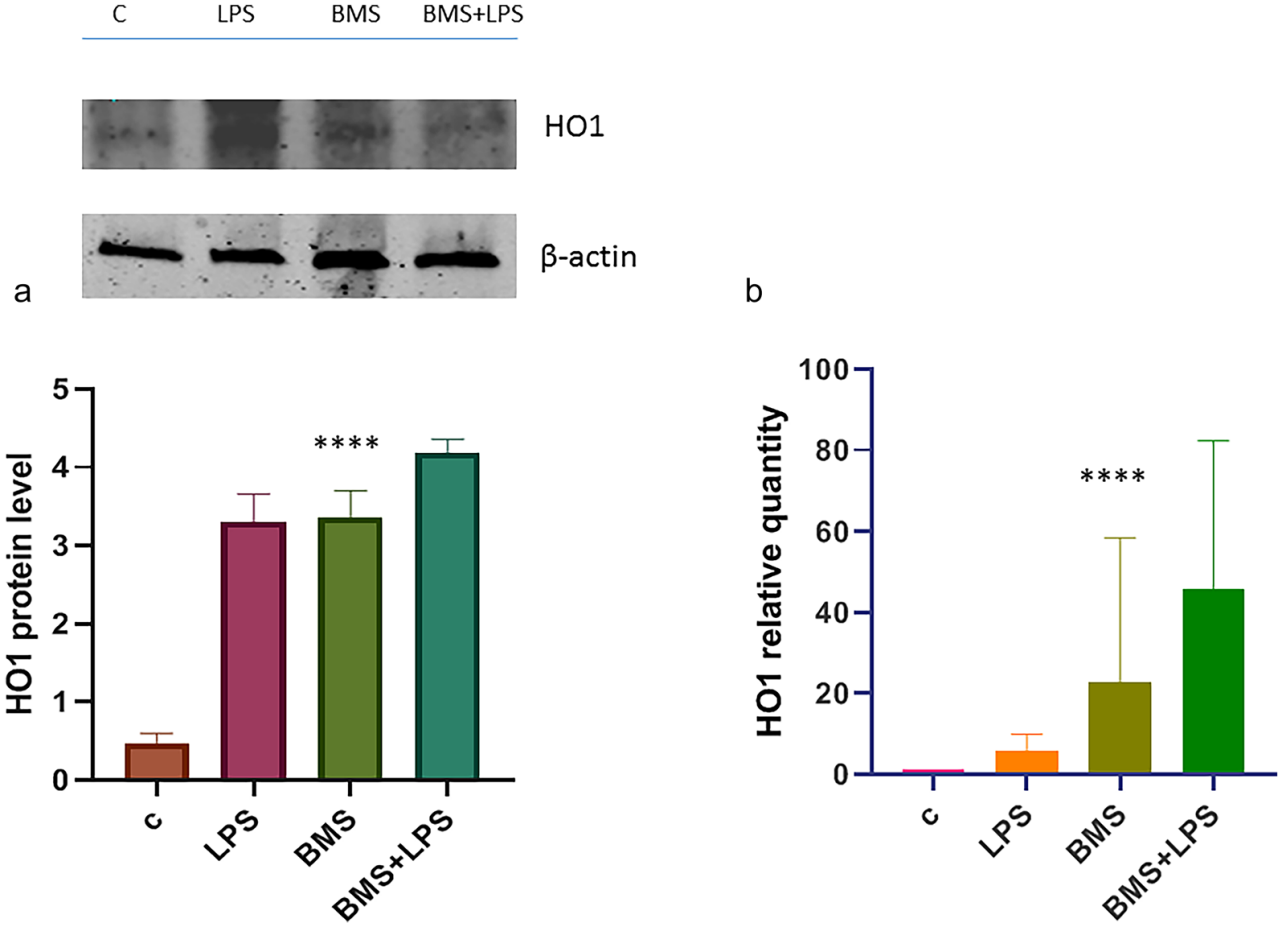
**a** Immunoblot analysis of HO1 protein in A549 cell line. A549 cells were treated with or without BMS-345541 1 µM for 24 h and then with LPS 100ngr/ml for 4 h. Total proteins were extracted and immunoblot analysis of HO1 protein level was effectuated. Densitometry analysis is shown in the graph after normalization with actin. The data shown represent three independent experiments.. Data are reported as the mean ± SD; asterisks (****) indicate significant differences among groups (**** p < 0.001), **b** Analysis of HO1 mRNA expression in A 549 cells. The cells were treated as above. Total RNA was extracted, and cDNA was analyzed by qPCR with HO1 primers. Non-stimulated cells worked as control. Levels are expressed in arbitrary units as fold increase compared to controls assumed as 1. The data shown represents three independent experiments, each of which performed in triplicate. Asterisks (*) indicate significant differences among groups (****p < 0.0001).

It is known that Heme Oxigenase-1 (HO1) is one of the most important enzymes in the antioxidant and anti-inflammatory response. The beneficial effect of HO1 during the inflammation onset is associated with the degradation of the heme group and with its anti-inflammatory products, biliverdin and CO. Therefore, HO-1 induction has a prominent role in the anti-oxidative response, and its induction is useful to contrast inflammatory processes. So, we analyzed transcript and protein levels by immunoblot and qPCR and the results are showed in Fig. 3. It is remarkable to notice that the BMS- 345,541 treatment induces an increase in the HO1 mRNA and protein level.

### HSP90 is Functionally Active in BMS-345541 Treated A549 Cell Line

**Fig. 4:**
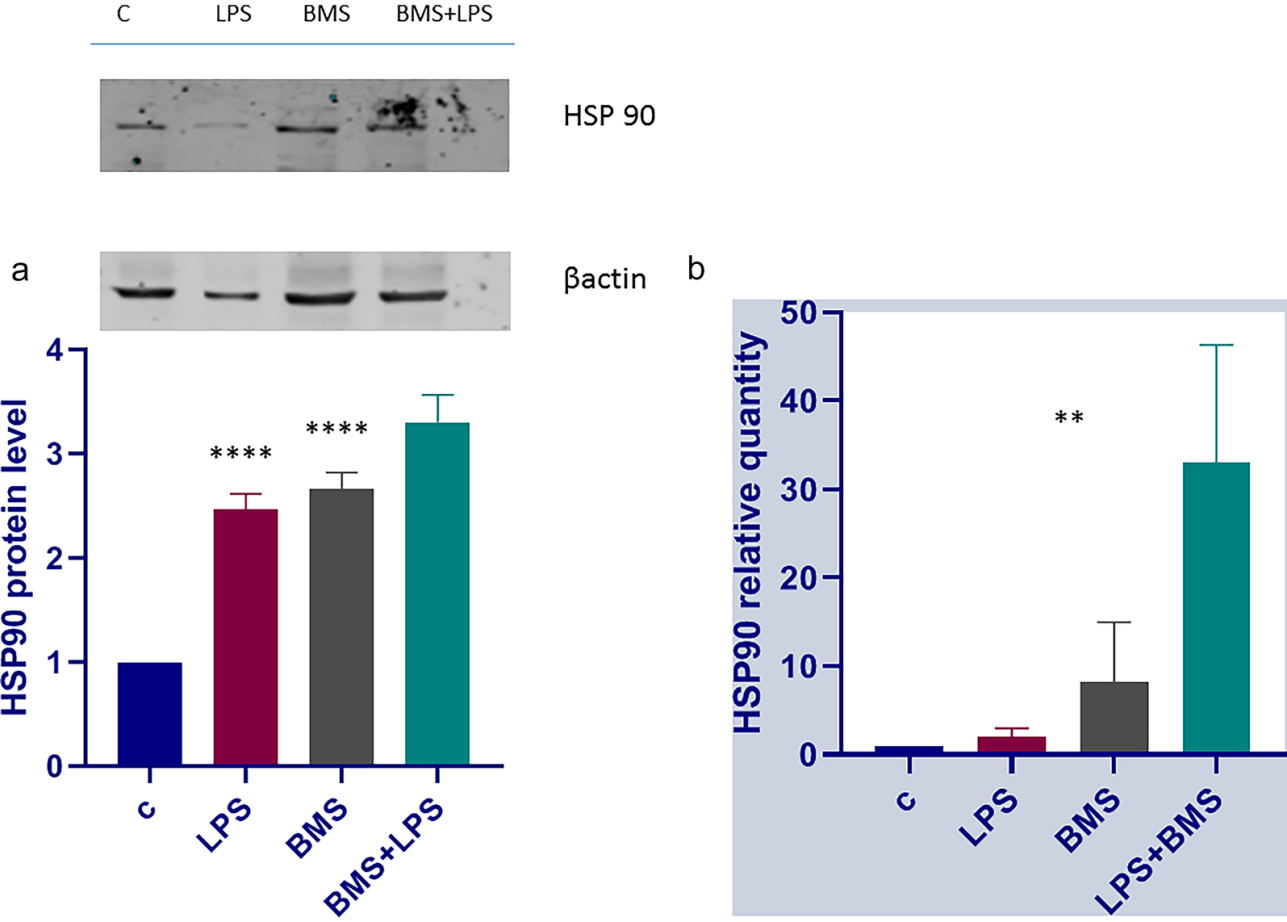
**a** Immunoblot analysis of HSP90 protein in A549 cell line. A549 cells were treated with or without BMS-345541 1 µM for 24 h and then with LPS 100ngr/ml for 4 h. Total proteins were extracted and immunoblot analysis of HSP 90 protein level was effectuated. Densitometry analysis is shown in the graph after normalization with actin. The data shown represent three independent experiments. Data are reported as the mean ± SD; asterisks (****) indicate significant differences among groups (**** p < 0.001), **b** Analysis of HSP90 mRNA expression in A549 cells. The cells were treated as above. Total RNA was extracted and cDNA was analyzed by qPCR. Non-stimulated cells worked as control. The difference between means is remarkable (p < 0.05). The data shown represent three independent experiments, each of which performed in triplicate. Levels are expressed in arbitrary units as fold increase compared to controls assumed as 1.

**Fig. 5:**
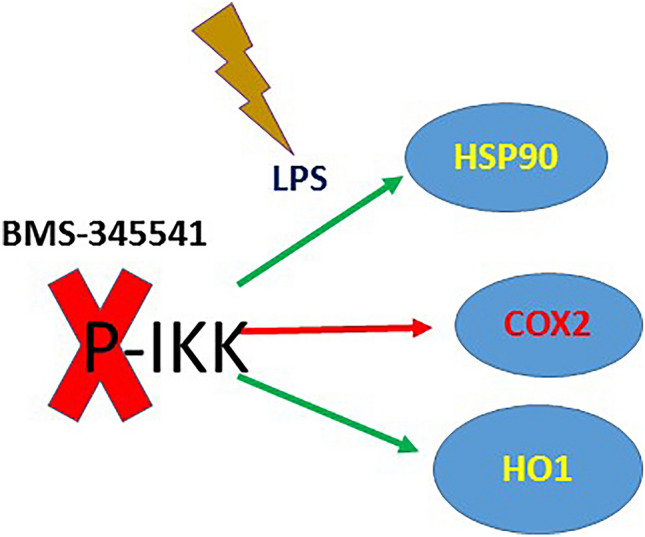
Representation of the effects induced by depletion of phosphorylation of IKK kinase in LPS treated A549 on the antioxidant pathway and HSP90 activity. Alterations in IKK kinase phosphorylation in A549 cell line treated with LPS do not induce COX2 activity, but significantly induce the HO1 and HSP90 activity.

One of the most important cytoprotective pathway is the heat shock response. Oxidative conditions and some chemicals are all factors that induce alteration of cellular homeostasis and cellular stress. The activity of HSP90 protein provides a powerful response helping cells to survive at injury, so it is extremely important that HSP90 mantains its functionality. Many previous studies have shown that HSP90 regulation depends on Keap1 and IKK; so we are studying the HSP90 activity both in an *in vitro* model of Keap1 loss of functionality and of BMS-345541-mediated IKK kinase inhibition. Total protein and RNA were extracted from A549 cells treated with (or without) 1 µM BMS-345541 and 100ngr/ml LPS for 4 h. Immunoblot and qPCR analyses were conducted, and results are shown in Fig. 4. The differences of HSP90 protein level in each group are significant. Figure 4a shows an increase in the HSP90 protein level in BMS-345541 and LPS treated A549 cells. To confirm this result we have conducted a qPCR analysis of HSP90 mRNA. The amplification of cDNA with specific primers for Hsp90 and HPRT as internal standard, showed that BMS-345541 and LPS treatment significantly triggers the Hsp90 mRNA transcription. These results are very important because they show that the HSP90 gene regulation is not closely tied up to IKK kinase and then blockage of the IKK phosphorylation cascade does not turn off the defence ability of the cells (Fig. 5).

## DISCUSSION

The present study revealed the cross-talk between the IKK kinase, Keap1 and heat shock and antioxidative pathways. We inhibited the phosphorylation of IKK kinase by using BMS- 345,541, a well-known IKK inhibitor, and then we analyzed the COX2, HO1 and HSP90 protein and mRNA level, as they are key enzymes in the cytoprotective network. The IKK kinase pathway is mainly involved in inflammation signaling and tumorigenesis: in fact, IKKα and IKKβ kinases are fundamental activators of these processes; specifically, IKK phosphorylation is responsible for the release of proinflammatory cytokines and the increase of mRNA levels of genes of inflammatory mediators [23, 24]. A widely applied strategy in the field of anticancer and anti-inflammatory therapies is the inhibition of long-term activation of the IKK complex through drugs and kinase inhibitor. BMS- 345,541 is a synthetic, well-known inhibitor of the catalytic subunits of IKK kinase, the one we used in our study to inhibit IKK phosphorylation. Our analysis was conducted in A549 cell line that is a suitable study model *in vitro*, as it is a tumour cell line owing to its point mutation in the Keap1 allele, an important regulator of the cell stress response system [18]. Many studies have pointed out that there exists a crosstalk between Keap1, IKK kinase and HSP90 [11, 12, 24–29]. There is still a lot to do to clarify the molecular mechanism underlying this interconnection and to develop anti-inflammatory and anticancer therapies which do not negatively affect the cell’s response to oxidative stress. BMS- 345,541 actively reduces the LPS-induced IKK phosphorylation in A549 cell line, and it is a useful *in vitro* model to proceed on the investigation of the effects of IKK inhibition on the two most important cytoprotective pathways. Pro-inflammatory activity is supported by COX-2 enzyme whose activation occurs when pro-inflammatory conditions take place [30]. The COX-2 enzyme—also known as prostaglandin-endoperoxide synthase (PTGS)—catalyses the formation of key biological mediators such as prostanoids (prostaglandins, prostacyclin and thromboxane). In our previous study we showed that Keap1 protein provides and supports the COX2 activity [26], here we show that in A549 cells, despite LPS stimulus, the inhibition of IKK activation induced by BMS- 345,541 produces an inhibition of COX2 activity. Furthermore, we analysed HO1activity, as it is essential to an effectiveness response to oxidative stress and represents a cytoprotective enzyme playing a crucial protective role, regulating important biological processes including inflammation, apoptosis, cell proliferation [31, 32]. The link between HO1 and the transcription factor Nrf2 is significant in cellular defense mechanisms. Nrf2 is a master regulator of the antioxidant response in cells. When cells are exposed to oxidative stress or harmful agents, Nrf2 gets activated and translocates to the cell nucleus. There, it binds to specific DNA sequences known as antioxidant response elements (AREs) in the promoter regions of target genes, including HO1.We highlighted that the molecular approach based on inhibition of IKK in A549 cells ensures the maintenance of the HO1 activity. Inflammation is closely related to the onset of oxidative stress and the activation of heat shock response. The results shown in this study demonstrate that the BMS- 345,541 is an inducer of the activity of HSP90 protein in A549 cells. This event implies an increased HSP90 gene and protein expression. HSP90 protein drives the heat shock response, performs multiple function and interacts with IKK kinase [27, 28, 33, 34]. The HSP90 activation induced by BMS- 345,541 highlights that HSP90 activity is not dependent on IKK phosphorylation. The inhibition of IKK phosphorylation is a very effective approach for treating the advance of inflammatory process. Plus, if considered that HSP90—one of the most important regulator of cellular homeostasis—keeps on maintaining its activity at the same time, it proves to be a successful result. The network Keap1-IKK-HSP90 is very interesting: it has lots of implications, being involved in inflammation, chemoresistance and cellular homeostasis [35–37].

## Data Availability

The data that support the findings of this study are available from the corresponding author, [G.M.], upon reasonable request.
